# Comparison of Three Nebulizer Nozzles Used During Pressurized Intraperitoneal Aerosol Chemotherapy Procedures in a Rabbit Model with Peritoneal Surface Malignancies: NOMOS Project

**DOI:** 10.1245/s10434-025-18002-4

**Published:** 2025-08-05

**Authors:** Abdelkader Taibi, Marie-Laure Perrin, Catherine Yardin, Julie Usseglio, Sylvaine Durand Fontanier, Sylvia M. Bardet

**Affiliations:** 1 CNRS, XLIM, UMR 7252, University Limoges, Limoges, France; 2 Visceral Surgery Department, Dupuytren University Hospital, Limoges, France; 3 EMIS Research, Dupuytren University Hospital, Limoges, France; 4 Cytology and Histology Department, Dupuytren University Hospital, Limoges, France; 5 CNRS, Institut de Recherche sur les Céramiques, UMR 7315, University Limoges, Limoges, France

**Keywords:** Peritoneal metastases, PIPAC, Nebulization nozzle, Animal model, Intraperitoneal chemotherapy, Oncological response

## Abstract

**Background:**

Pressurized intraperitoneal aerosol chemotherapy (PIPAC) improves local drug delivery and has shown promising results for treating peritoneal surface malignancies (PSMs); however, comparative data on the performance of different PIPAC nozzles in disease-bearing models are limited.

**Objective:**

This study aimed to evaluate the therapeutic efficacy of three PIPAC nozzles in a rabbit model of PSM.

**Methods:**

Nine immunocompetent New Zealand rabbits received intraperitoneal injections of VX2 tumor cells to induce PSM. The animals were randomized into three groups (*n* = 3) and treated with oxaliplatin-based PIPAC using CapnoPen^®^, HurriChem™, or MCR-4 TOPOL^®^ nozzles on days 8, 15, and 21. Evaluations included the Peritoneal Cancer Index (PCI), tumor weight, histopathology (Peritoneal Regression Grading Score [PRGS]), imaging, and markers of proliferation (Ki-67) and apoptosis (TUNEL). The final results, including autopsy, were obtained on day 26.

**Results:**

CapnoPen^®^ showed a trend toward improved tumor regression, with significantly lower PCI scores at euthanasia. After the third PIPAC, the mean PCI was lower in the CapnoPen^®^ group compared with MCR-4 TOPOL^®^ (6.33 vs. 13.3; *p* = 0.041). Tumor weights were also lowest in the CapnoPen^®^ group. Mean PRGS values were 2.14 (CapnoPen^®^), 2.75 (HurriChem™), and 3.49 (MCR-4), with a significant difference between CapnoPen^®^ and MCR-4 (*p* = 0.033). All devices significantly reduced PCI compared with controls from a previous study (no chemotherapy: 21.6; *p* < 0.01).

**Conclusion:**

This initial study suggests that the type of PIPAC nebulizer may influence therapeutic efficacy in a rabbit model with peritoneal metastasis, highlighting the need for further controlled studies to confirm these preliminary findings

Peritoneal metastases (PMs) and peritoneal surface malignancies (PSMs) differ from other metastatic sites due to their unique pathophysiology, and are characterized by limited chemosensitivity, primarily caused by the poor diffusion and penetration of intravenous chemotherapy into peritoneal tissues.^1^

For patients with PMs, the benefits of intraperitoneal chemotherapy administration are supported by preclinical and pharmacokinetic data.^2,3^ To enhance the effectiveness of intraperitoneal chemotherapy, pressurized intraperitoneal aerosol chemotherapy (PIPAC) has been employed for over a decade by specialized PM teams.^4,5^ This innovative technique directly administers chemotherapy into the peritoneal cavity, providing superior tolerance (grade 3 or higher adverse effects < 10%), reduced cumulative toxicity, and improved histological responses.^4^ A further advancement in this domain is the development of PIPAC (which can be combined with systemic chemotherapy regimens such as LV5FU2, either alternately or simultaneously.^6^

Historically, the CapnoPen^®^ has been the most widely used medical device for over a decade. This laparoscopic nebulization nozzle, connected to a high-pressure injector, delivers intraperitoneal chemotherapies such as doxorubicin, cisplatin, and oxaliplatin. However, in 2022, reports of incidents and the emergence of different nozzles spurred the medical community to investigate alternative options. These discussions focused on evaluating spray nozzles based on parameters such as droplet size and spray angle to improve treatment outcomes.^7^ Medical devices undoubtedly play a central role in improving the therapeutic response and therefore, ultimately, the survival of patients treated for MSP.

To date, no study has assessed the performance of PIPAC spray nozzles using an animal model with PMs while employing objective efficacy criteria routinely applied in clinical practice. The aim of this experimental study was to compare the effectiveness of the most commonly used PIPAC spray nozzles in an immunocompetent rabbit model with PSM.

## Methods

This experimental study aimed to compare the effects of oxaliplatin PIPAC on tumor progression using three different nebulizer nozzles (CapnoPen^®^, HurriChem™, MCR-4 TOPOL^®^) in a rabbit model with PSM.

### Animal Selection

Four-month-old female New Zealand rabbits (weighing approximately 2–3 kg) were used (Centre d'élevage de Gontran Achard Pour La Vente Sous Contrôle Sanitaire [CEGAV SSC], Argenvilliers, France). The rabbits were maintained on a standard diet under a 12-h light/dark cycle. The animals followed a 1-week acclimation period and were euthanized and immediately autopsied at the end of the experiment. This study obtained ethical approval from the French Ministry of Research (Autorisation de Projets à But Scientifique #28502-2020120314287244), in compliance with ethical rules (decree n° 2001-131, European directive 86-609-CEE 1986) and the ARRIVE guidelines 2.0.^8^

### Experimental Protocol for Pressurized Intraperitoneal Aerosol Chemotherapy in Rabbits

The PIPAC procedure for rabbits was adapted from clinical treatment protocols^6^ and was performed as published in a previous study.^9^ Anesthesia was initiated via intramuscular injection using a combination of ketamine (Renaudin Laboratory, Itxassou, France) at a dose of 15–20 mg/kg, xylazine (Rompun 2%, Elanco, Cuxhaven, Germany) at 4 mg/kg, and acepromazine (Calmivet, Vetoquinol, Tarare, France) at 0.85 mg/kg. Prior to the procedure, the abdominal region was shaved and disinfected. A laparoscopic technique was employed, beginning with the insertion of a 12 mm balloon trocar under direct visual control. After establishing a stable pneumoperitoneum at 8 mmHg with carbon dioxide, an optical camera was used to inspect the abdominal cavity.

A second trocar, measuring 10 mm in diameter, was inserted at the midline and securely positioned. A nebulizing nozzle was connected to a high-pressure injector through a specialized conduit and introduced into the abdominal cavity via the trocar. The system’s integrity was verified by confirming no carbon dioxide leakage. The injector was then activated, with the nozzle configured for a controlled flow rate and a maximum pressure in line with manufacturer recommendations.

### Rabbit Model of Peritoneal Surface Malignancy

First, the VX2 tumor was obtained in two New Zealand White rabbits by implanting tumor fragments into the hind limb, in accordance with previously described methods.^9^ After a 15-day incubation period, the tumors were dissociated into a cell suspension, which was then delivered to multiple intraperitoneal locations, including the stomach surface, omentum, visceral peritoneum of the small bowel, and parietal peritoneum via a mini-laparotomy. Injections were performed using an 18-gauge spinal needle (Becton Dickinson, Franklin Lakes, NJ, USA). Tumors were allowed to develop for 8 days prior to the initiation of therapeutic interventions.

### Study Design

The animals were divided into three groups of five rabbits and randomized on day 8 after tumor implantation:

- 1 group: CapnoPen^®^, Capnomed, Zimmern, Germany
- 1 group: HurriChem™, ThermaSolutions, White Bear Lake, MN, USA
- 1 group: MCR-4 TOPOL^®^, SKALA-Medica, Sobĕslav, Czech Republic

Based on a 3.5 kg rabbit body surface area (0.233 m^2^), the rabbits were treated by PIPAC with 92 mg/m^2^ oxaliplatin. PIPAC procedures were conducted on days 8, 15, and 21 post-tumor implantations, with euthanasia on day 26.

**Fig. 1.**
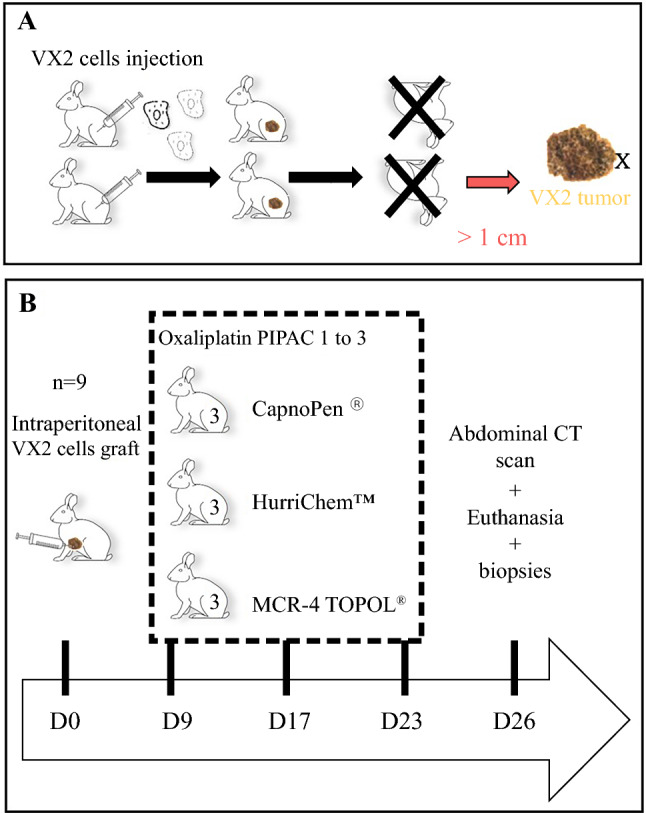
Timeline and experimental procedures of PIPAC in the rabbit for **a** obtaining the implantable VX2 tumors. **b** Oxaliplatin PIPAC study with the three nozzles. *PIPAC* pressurized intraperitoneal aerosol chemotherapy, *CT* computed tomography

The nebulizing nozzles were either purchased from Capnomed or supplied free of charge by the other companies (ThermaSolutions and SKALA-Medica) (Fig. 1).

### Evaluation Criteria of Tumor Evolution

Animal well-being was evaluated using a composite scoring system, which included assessments of mobility, facial cues, posture at rest, coat condition, nutritional and hydration status, and responsiveness to external stimuli (scale from 0 to 3 for each parameter). Welfare scores were documented prior to each PIPAC session. The weight of each animal was monitored daily throughout the entire procedure.

During each PIPAC, ascites volume was quantified and the Peritoneal Cancer Index (PCI) was determined by direct visual inspection by an expert surgeon. Tumor biopsies were obtained from a minimum of three distinct nodules,^10^ fixed in 4% paraformaldehyde, and paraffin-embedded for histological processing.

Sections of 4 µm thickness were stained with hematoxylin-eosin-safran (HES) using an automated BenchMark XT system (Roche). Further analyses included:

- immunohistochemical labeling with anti-Ki-67/MKI67 antibody (clone 8D5, NBP2-22112, Biotechne, France);
- TUNEL assay for apoptosis detection (HRP DAB, ab206386, Abcam, France)

The computed tomography (CT) scan with injection of contrast medium was performed before PIPAC#1 and after PIPAC#3 in order to compare across all groups:

- the mean of the sums of the size of the visualizable MP after the third PIPAC;
- the thickness of the parietal lateral wall (abdominal internal and external oblique muscles + transversal abdominal muscle).

The histological response was graded using the Peritoneal Regression Grading Score (PRGS) according to Solass et al.^11^ A surface-based index representing proliferation and apoptosis rates (%) was calculated from Ki-67 and TUNEL staining using Fiji/ImageJ (National Institutes of Health). Tumor weights after the third PIPAC were compared across experimental groups on day 26, at the end of the study.

### Complementary Study

To complement the current analysis, the present PCI results of the three nozzle groups were compared with those of an untreated control group (receiving physiological saline instead of oxaliplatin chemotherapy), extracted from a previously published study conducted under comparable experimental conditions (same number of PIPAC procedures, similar tumor sizes and cell types, etc.).^9^ This approach was adopted in accordance with the 3Rs principle to minimize the number of animals used.

### Statistical Analysis

Data were analyzed using a one- or two-way repeated measure of analysis of variance (ANOVA), with post hoc tests to locate the source of significant differences using Bonferroni or Sidak corrections for multiple comparisons (GraphPad Prism). The results are presented as means with standard error (± standard deviation [SD]). Asterisks in the figures indicate statistically significant differences compared with controls where the probability of falsely rejecting the null hypothesis was < 5% (*p* < 0.05). The surgeon and the pathologist were blinded during the assessment of the analyzed endpoints.

## Results

### Animal Well-Being Score

The well-being score was stable until the end of PIPAC#2 in all groups. This score slightly increased between 1 and 3 points over 18 in the three groups, with no statistically significant difference.

### Radiological Response

**Fig. 2.**
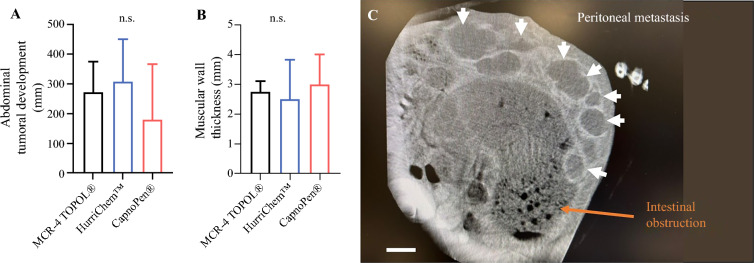
Radiological response after the third oxaliplatin PIPAC in the three groups. **a** Abdominal tumoral development (means ± SD); **b** muscular wall thickness (means ± SD); **c** abdominal CT scan image from a rabbit (white arrows show extensive VX2 peritoneal metastasis disease, and the orange arrow shows an intestinal obstruction example). *PIPAC* pressurized intraperitoneal aerosol chemotherapy, *SD* standard deviation, *CT* computed tomography, *n.s.* non-significant

As shown in Fig. 2a, the mean total size of visualizable PMs after the third PIPAC was lower in the CapnoPen^®^ group (180.96 mm ± 186.4) compared with the MCR-4 TOPOL^®^ group (272.73 mm ± 102.5) and the HurriChem™ group (309.1 mm ± 142.4); however, these differences were not statistically significant [F(2,5) = 0.5345; *p* = 0.6161]. This trend was further supported by the analysis of muscle cachexia, as muscle wasting was stronger in the HurriChem™ group (2.5 mm ± 1.32), whereas rabbits in the CapnoPen^®^ group (3 mm ± 1) exhibited a tendency toward reduced muscle loss (Fig. 2b, c); however, this difference was not statistically significant [F(2,5) = 0.1667; *p* = 0.8510].

### Peritoneal Cancer Index (PCI) and Tumor Weight

**Fig. 3.**
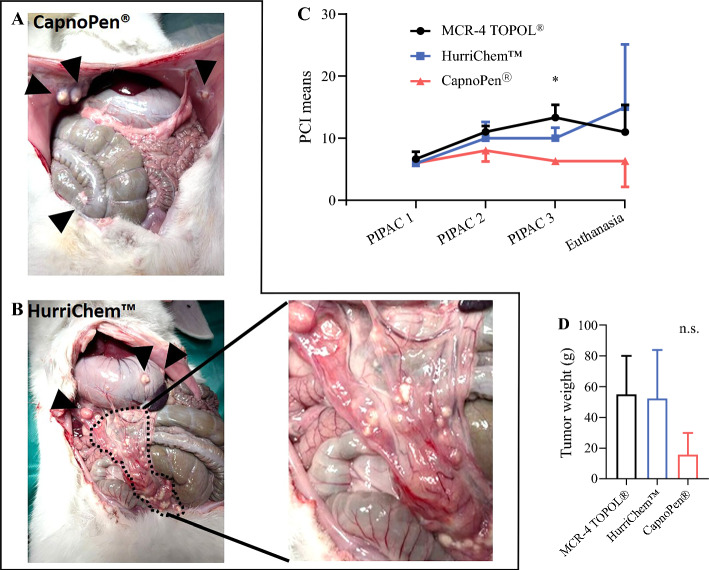
Laparotomy following the third oxaliplatin PIPAC illustrating **a** low and **b** high PCI. **c** The evolution of PCI across the PIPAC procedures. **d** Residual tumor weights measured at euthanasia. *p < 0.05. *PIPAC* pressurized intraperitoneal aerosol chemotherapy, *PCI* Peritoneal Cancer Index, *n.s.* non-significant

Following the second oxaliplatin PIPAC, a non-significant trend toward a lower PCI was observed in the CapnoPen^®^ group compared with the other two groups (Fig. 3). This difference became statistically significant after the third PIPAC, particularly when comparing the CapnoPen^®^ group with the MCR-4 TOPOL^®^ group (mean PCI 6.33 vs. 13.3; *p* = 0.041).

The evolution of PCI also varied between groups. In the CapnoPen^®^ group, the PCI remained relatively stable with minimal variability throughout the treatment period (PIPAC#1: 6 ± 0; PIPAC#2: 8 ± 1.73; PIPAC#3: 6.33 ± 0.58; euthanasia: 6.33 ± 4.16; non-significant). In contrast, the PCI tended to worsen after the third PIPAC in the HurriChem™ group (PIPAC#1: 6 ± 0; PIPAC#2: 10 ± 2.65; PIPAC#3: 10 ± 1.73; euthanasia: 15 ± 10.15; non-significant), whereas in the MCR-4 TOPOL^®^ group, an initial increase at the second PIPAC was followed by a further rise at the third PIPAC and a slight decrease at euthanasia (PIPAC#1: 6.67 ± 1.15; PIPAC#2: 11 ± 1; PIPAC#3: 13.33 ± 2.08; euthanasia: 11 ± 4.36; non-significant).

The overall statistical analysis using two-way ANOVA revealed a significant effect of the number of PIPAC procedures on tumor progression [F(1.19, 7.15) = 15.5; *p* = 0.004]. Additionally, a statistically significant effect of the nozzle type was observed [F(2,6) = 5.95; *p* = 0.038].

These findings were corroborated by measurements of residual tumor weight at euthanasia, which were lower in the CapnoPen^®^ group (15.8 g ± 14.1) following oxaliplatin-based PIPAC, compared with the MCR-4 TOPOL^®^ (55.1 g ± 25) and HurriChem™ (52.5 g ± 31.5) groups.

### Histological Response, Cell Proliferation, and Cell Death Analysis

**Fig. 4.**
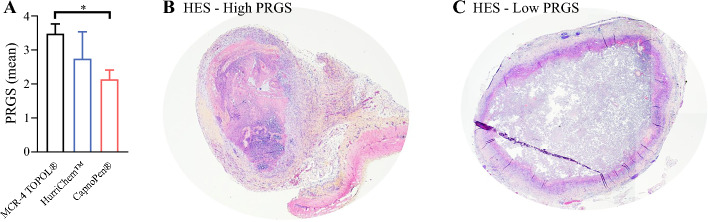
**a** PRGS evaluated in peritoneal tumor samples after the third PIPAC across all groups. HES illustrates the differences in histological regression: **b** high PRGS, indicating poor regression; **c** low PRGS, consistent with marked tumor regression. * *p* < 0.05. *PRGS* Peritoneal Regression Grading Score, *PIPAC* pressurized intraperitoneal aerosol chemotherapy, *HES* hematoxylin and eosin staining

The mean PRGS was lower in the CapnoPen^®^ group compared with the other two groups, with a statistically significant difference observed at euthanasia (one-way ANOVA based on nozzle type: F(2,6) = 5.402; *p* = 0.0455) (Fig. 4). Specifically, the CapnoPen^®^ group showed a mean PRGS of 2.14 ± 0.27 versus 3.49 ± 0.28 in the MCR-4 TOPOL^®^ group and 2.75 ± 0.78 in the HurriChem™ group. Post hoc analysis revealed a significant difference between the CapnoPen^®^ and MCR-4 TOPOL^®^ groups (*p* = 0.0333). Moreover, a complete or major histological response was observed in 2/3 rabbits (66%) in the CapnoPen^®^ group compared with 1/3 rabbits (33%) in the HurriChem™ group and none in the MCR-4 TOPOL^®^ group (0%).

Cell proliferation, assessed by Ki-67 immunohistochemistry in tumor samples collected at euthanasia, confirmed the antiproliferative effect of oxaliplatin-based PIPAC across all groups, regardless of the nozzle type, with a percentage of Ki-67-positive cells below 1% (data not shown). Cell death by TUNEL assessment did not show any differences between the groups (data not shown).

### Complementary Study: Comparison of PCI Between Nozzle Groups and an Untreated Control Group

**Fig. 5.**
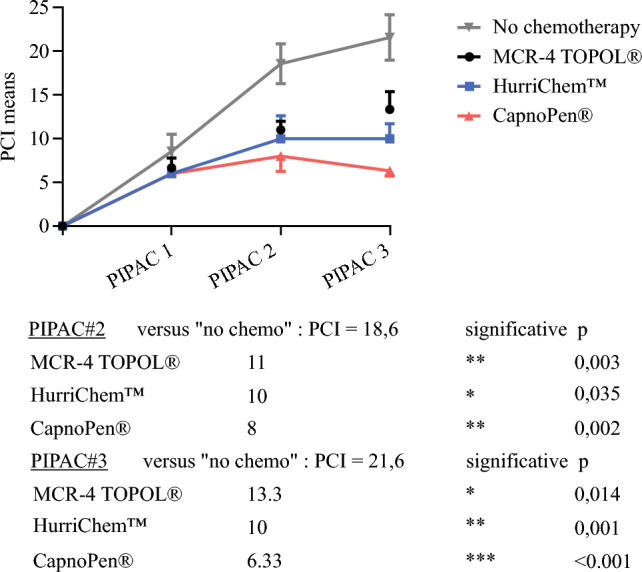
Evolution of the PCI across PIPAC procedures. Data for the three nozzle groups (oxaliplatin-based PIPAC) are derived from Fig. 3c, while data for the non-treated control group (no chemotherapy, *n* = 5) were extracted from a previously published study.^9^ Two-way ANOVA revealed a significant effect of the number of PIPAC procedures [F(2.03, 20.3) = 167; *p* < 0.001] and a significant effect of the treatment group (three nozzle types and untreated control) [F(3, 10) = 74.9; *p* < 0.001]. *PCI* Peritoneal Cancer Index, *PIPAC* pressurized intraperitoneal aerosol chemotherapy, *ANOVA* analysis of variance

To strengthen the current analysis, PCI results for the three nozzle groups were compared with control data (physiological saline) from a prior study performed under comparable conditions.^9^ All three medical devices demonstrated a significant impact on tumor regression, as assessed by the PCI, beginning with the second PIPAC. At PIPAC#2, the mean PCI was markedly lower in all treated groups compared with the non-treated control group (no chemotherapy: 18.6 ± 2.3; MCR-4: 11 ± 1; HurriChem™: 10 ± 2.65; CapnoPen^®^: 8 ± 1.73) [Fig. 5]. This effect became even more pronounced at PIPAC#3, with PCI values further diverging while remaining statistically different from the control group (no chemotherapy: 21.6 ± 2.61; MCR-4: 13.33 ± 2.08; HurriChem™: 10 ± 1.73; CapnoPen^®^: 6.33 ± 0.58).

## Discussion

This experimental study demonstrates that PIPAC using oxaliplatin leads to substantial tumor control across all three nozzle technologies evaluated (CapnoPen^®^, HurriChem™, and MCR-4 TOPOL^®^), with notable differences in performance. CapnoPen^®^ consistently showed favorable outcomes in radiological, macroscopic, and histological assessments. Although not all differences reached statistical significance, a consistent trend favored CapnoPen^®^, particularly regarding lower tumor burden on imaging, reduced muscle cachexia, and stable PCI throughout treatment.

Comparing the three nozzles without a non-treated control group limits objective interpretation of treatment efficacy due to a potential magnifying effect. Including a control group provides an essential perspective and reduces the risk of overinterpreting minor differences. At PIPAC#3, the PCI remained strikingly high in non-treated animals, underscoring the strong antitumor effect of PIPAC. Two-way ANOVA confirmed significant effects of both the number of PIPACs and the nozzle type on tumor progression. The control group helped contextualize nozzle performance and ensured a more balanced evaluation.

The original medical device used for over 10 years is the CapnoPen^®^, a laparoscopic nebulization nozzle for liquids, connected to a high-pressure injector; this was the only clinically available option. The technical and clinical performance of the original CapnoPen^®^ nozzle has been extensively studied in both preclinical and clinical contexts; however, research teams have encountered a triple issue:

- the recent introduction of new nebulization devices without a thorough evaluation of their physical properties by independent research teams (not affiliated with manufacturers);^12–15^
- the lack of clinical data on large populations for each device;
- the absence of comparative studies between the different nozzles.

This situation has introduced confounding bias into clinical studies as we do not know whether the new spray nozzles are equivalent to the original technology that may influence oncological outcomes. This experience led the medical community to (1) promote understanding about the issue of PIPAC medical devices;^16^ (2) establish specific recommendations for spray nozzles;^7^ and (3) initiate a broader discussion and the need to test and compare intraperitoneal chemotherapy spray nozzles beyond conventional parameters such as droplet size and spray angle.

Two teams have recently published studies comparing medical devices based primarily on technical criteria. The first, by Göhler et al. (Table 1^17^), compared four nebulizers on several technical aspects; however, the methodology of this study shows some weaknesses. The authors assessed the chemical resistance of the nozzle material by exposing it to a cytostatic solution for 12 h, then storing it in a dry, dark place at room temperature for 12 days; however, in clinical practice, the nozzle is in contact with chemotherapy for approximately 36 min (6 min of spraying and 30 min of exposure). Moreover, the dose of chemotherapy used in the study does not reflect clinical reality.

Pocard et al., compared two spray nozzles: CapnoPen^®^ (CAPNOMED) and Nebulo^®^ (GAMIDA), in two groups of sheep (*n* = 3). There was no significant difference in tissue penetration depth between the groups. Regarding the peritoneum, 40% showed a penetration depth >100 μm in the CapnoPen^®^ group compared with 5% in the Nebulo^®^ group (*p* = 0.06); however, this study was conducted using only doxorubicin in animals without disease.^18^

The droplet size distribution, spray cone angles, spray morphology, and drug deposition distribution are essential criteria that have been widely compared in several studies.^17^

The results obtained in our study can be interpreted according to several parameters. The intra-abdominal pressure applied was lower (8 mmHg) in rabbits than that typically used in clinical practice in humans. This choice was made in agreement with the well-being committee to avoid pressure-related complications in the rabbit model. This lower pressure may likely partly explain the outcomes observed with the Topol and HurriChem nozzles. However, it did not appear to negatively impact the results obtained with the CapnoPen, suggesting that this nozzle remains effective even at reduced operating pressure. Second, the physical characteristics of the nozzles used may have influenced the distribution and efficacy of the aerosol. The MCR-4 Topol, for instance, is characterized by a larger orifice and produces larger droplets with a very wide spray cone, but requires higher flow rates to ensure stable nebulization. In contrast, the CapnoPen^®^ and HurriChem™ nozzles have smaller orifices, generate finer droplets, and maintain a wide and stable spray cone even at lower or moderate flow rates.^19^ These technical differences may affect aerosol dispersion within the peritoneal cavity and could account for some of the variations observed in therapeutic response between the devices. The interval between PIPAC procedures in our study (1 week) differs from the 6-week interval used in clinical settings. This adjustment was necessary due to the limited lifespan of the rabbit model. While this difference might raise concerns regarding the comparability to clinical protocols, our previous publication using the same model has demonstrated both its feasibility and therapeutic relevance.^9^ It does not appear to be an obstacle to the effectiveness of PIPAC performed with oxaliplatin or the combination of cisplatin and doxorubicin. The main limitation of our experimental study was the small sample size per group due to ethical considerations, a common challenge in *in vivo* research. However, we have demonstrated that all three nozzles are effective tools for delivering chemotherapy via PIPAC, with CapnoPen^®^ showing a notably stronger advantage.

## Conclusion

This experimental study showed that nozzle design may influence oncological response in a rabbit model with PSM, potentially accelerating the translation of these findings to humans. The results emphasize the importance of investigating nozzle design as a factor in PIPAC efficacy and highlight the need for dedicated comparative clinical studies to optimize treatment delivering and patient outcomes.
